# Northernmost (Subarctic) and deepest record of *Paleodictyon*: paleoecological and biological implications

**DOI:** 10.1038/s41598-023-34050-w

**Published:** 2023-05-03

**Authors:** Olmo Miguez-Salas, Francisco J. Rodríguez-Tovar, Allan A. Ekdale, Stefanie Kaiser, Angelika Brandt, Andrew J. Gooday

**Affiliations:** 1 Department of Marine Zoology, Senckenberg Research Institute and Natural History Museum, 60325 Frankfurt, Germany; 2 Departamento de Estratigrafía y Paleontología, Universidad de Granada, Av. Fuentenueva, 18002 Granada, Spain; 3 Department of Geology and Geophysics, University of Utah, Lake City, UT 84112 USA; 4 Department of Biological Sciences, Institute of Ecology, Evolution and Diversity, Johann Wolfgang Goethe University Frankfurt, Max-Von-Laue-Str. 13, 60438 Frankfurt, Germany; 5 National Oceanography Centre, Southampton, European Way, Southampton, UK; 6 Life Sciences Department, Natural History Museum, Cromwell Road, London, SW7 5BD UK

**Keywords:** Ocean sciences, Palaeoceanography, Behavioural ecology, Biogeography, Palaeoecology

## Abstract

*Paleodictyon* is one of the most iconic and widespread of trace fossils in the geological record. However, modern examples are less well known and restricted to deep-sea settings at relatively low latitudes. Here, we report the distribution of *Paleodictyon* at six abyssal sites near the Aleutian Trench. This study reveals for the first time the presence of *Paleodictyon* at Subarctic latitudes (51°–53°N) and at depths over 4500 m, although the traces were not observed at stations deeper than 5000 m suggesting that there is some bathymetric constraint for the trace maker. Two small *Paleodictyon* morphotypes were recognized (average mesh size of 1.81 cm), one having a central hexagonal pattern, the other being characterized by a non-hexagonal pattern. Within the study area, *Paleodictyon* shows no apparent correlation with local environmental parameters. Finally, based on a worldwide morphological comparison, we conclude that the new *Paleodictyon* specimens represent distinct ichnospecies that are associated with the relatively eutrophic conditions in this region. Their smaller size may reflect this more eutrophic setting in which sufficient food can be obtained from a smaller area in order to satisfy the energetic requirements of the tracemakers. If so, then *Paleodictyon* size may provide some assistance when interpreting paleoenvironmental conditions.

## Introduction

*Paleodictyon* Meneghini, 1850^1^ is a well-known trace fossil belonging to the graphoplyptid group, characterized as a “three-dimensional burrow system consisting of horizontal net composed of regular to irregular hexagonal meshes and vertical outlets. Preferentially the net is preserved”^2^ (emended diagnosis by Uchman^3^). Regular nets of *Paleodictyon* first appear in the Early Cambrian^4^ and are found in modern oceans^5–9^. Mesh size and tunnel diameter, the basic ichnotaxobases used for distinguishing ichnospecies of *Paleodictyon*, show different size trends from the Paleozoic to the Neogene^10^. In the fossil record the traces are mainly associated with deep-sea flysch deposits, but they have also been reported occasionally in shallower-water deposits^3,11^.

Compared to *Paleodictyon* trace fossils*,* which are common and well-studied, observations on modern examples are relatively recent and there are only a few detailed studies of *Paleodictyon* observed in deep-sea bottom photographs^5,12^. This is in part because it is difficult and expensive, in terms of both time and cost, to undertake ichnological analyses in deep-sea environments. In the fossil record 32 ichnospecies of *Paleodictyon* have been distinguished^3^, whereas modern examples are referred to only two ichnospecies. The most common of the two modern ichnospecies is *Paleodictyon nodosum* Seilacher, 1977, characterized by rows of openings that intersect at an angle of 120° and presumably represent the openings of tubes extending up from the nodes of the underlying horizontal hexagonal honeycomb network of tunnels, located 2–3 cm below the sediment surface^5^. The other modern ichnospecies, *Paleodictyon tripatens*, has a less regular surface pattern because the vertical openings are located on three of the six sides of the horizontal hexagonal network in the sediment^3,13^.

Modern examples of *Paleodictyon*, particularly those of *P. nodosum*, are widely distributed in deep-sea sediments (see Fig. 1 in Gerdes et al., 2021). They are reported in the North Atlantic along the Mid-Atlantic Ridge (MAR)^5,12^, in the South Atlantic^14^, and in the Pacific along the Australian margin^6^, as well as in western^15^ and equatorial regions (Clarion Clipperton Fracture Zone [CCZ])^7^ and recently on the southern central Indian Ridge and the Southeast Indian Ridge^9^. All these records are from tropical or subtropical areas close to the Tropics of Cancer (23.5°N) and Capricorn (23.5°S), and there are no published records from beyond 50°N and 50°S. Modern *Paleodictyon* are confined to the deep sea, at water depths from 1400 to around 4000 m^9^. Neoichnological studies have failed to reveal any direct evidence regarding the nature of the organism responsible for the traces, while paleoichnological studies have not clarified its paleoenvironmental requirements. Despite the persistent mystery, it has been speculated that two taxa, hexactinellid sponges and xenophyophores, are the most likely tracemakers, although in the absence of substantial evidence for either of these candidates^5^, we regard the *Paleodictyon* tracemaker as unknown. Recently, the functional morphology of *Paleodictyon* has been tested using a computational fluid dynamics approach. This suggests that the tracemaker, whatever its identity, constructs the three-dimensional hexagonal tunnel network in a way that creates a balance between the efficiency of ventilation and physical stability against erosion^16^.

**Figure 1:**
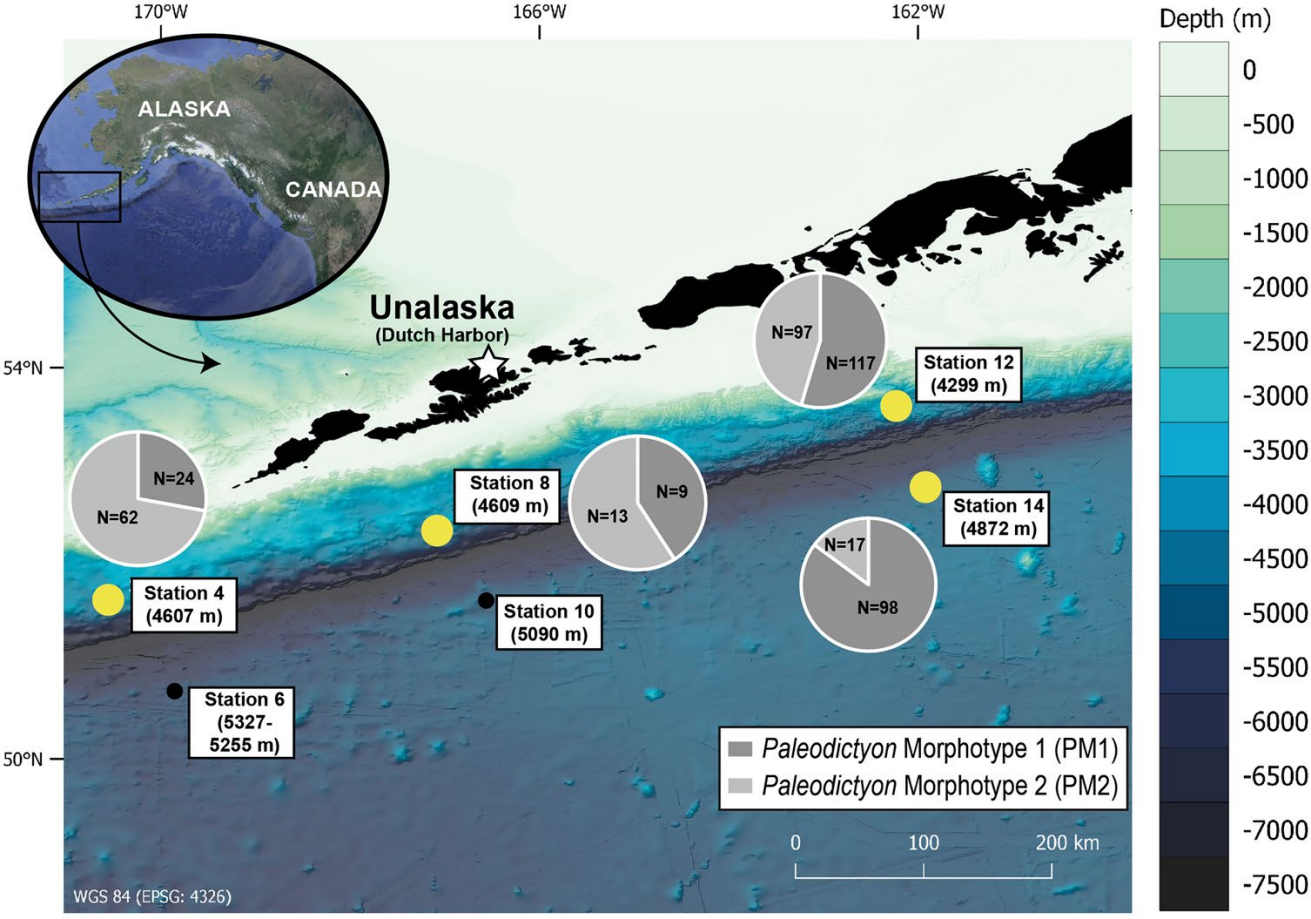
Bathymetric map of the study area near the Aleutian Trench with the locations of the stations where the OFOS was deployed (courtesy of Dr. Anne-Cathrin Wölfl and Kevin Kess). Stations designated by thick yellow dots indicate appearance of *Paleodictyon*, and stations designated by small black dots indicate absence. Pie chart diagrams illustrate the proportion of *Paleodictyon* Morphotype 1 (PM1) vs. *Paleodictyon* Morphotype 2 (PM2) at the different stations. Note that the proportion of PM1 becomes greater toward the east.

New discoveries in the fossil record, and especially on modern examples, are required in order to advance our understanding of *Paleodictyon,* and in particular to decipher the relationship between *Paleodictyon* and environmental variables at a global scale. Here, we report, for the first time, abundant modern *Paleodictyon* at sites above 50°N latitude in a Subarctic environment and at over 4500 m depth. This is the northernmost and the deepest unambiguous record to date. The aims of this study are (a) to describe variations in the morphological patterns of *Paleodictyon* identified on the abyssal plains across the Aleutian trench, (b) to evaluate possible environmental constraints on the distribution of these Aleutian variants, and (c) to assess environmental factors that may influence the distribution and morphology of *Paleodictyon* morphotypes on a global scale.

## Material and methods

This study is based on data acquired during the ‘AleutBio’ expedition aboard the German research vessel R/V *SONNE* (cruise SO293; July–September 2022), whose overall objective was to investigate the biogeography and biodiversity of deep-sea biota across the Bering Sea and Aleutian Trench region. The analyses of seafloor imaging was undertaken using the Ocean Floor Observation System (OFOS), a towed camera that is part of the shipboard equipment of the R/V *SONNE.* This system is equipped with a Full-HD video camera and a 45 megapixel mirrorless camera (Canon EOS R5; resolution of 8192 × 5464 pixels). Three laser-points arranged in a triangle and separated by 40 cm distances provide a scale, calibrated for the still camera. Six OFOS transects sampled the abyssal seafloor near the Aleutian Trench at depths between 4299 and 5327 m (OFOS cannot be deployed below 6000 m) (Fig. 1). The seafloor sediment was mainly composed of diatoms and radiolarians mixed with muddy terrigenous clay. Each camera transect covered more than 1 km with an average visible width of 1.5 m, resulting in a survey of more than 15,000 m^2^ of seafloor (Table 1). Approximately one still image was obtained every 10 s of the transect, depending on flash charge and focus conditions, resulting in a total of more than 5000 still images (Table 1). The visible area of each still image was limited by the domed housing of the camera system, which provides a circular in-focus area at the centre of the image. Thus, since *Paleodictyon* patterns have a millimetric scale, only specimens within the in-focus area were considered for further morphological analysis*.* Following calculations in Sigwart et al.^17^ of the in-focus area of each still image, an average of 60.5% of the frame was considered for calculating *Paleodictyon* densities.

**Table 1:** Stations location and OFOS transects at the Aleutian Trench nearby abyssal area including information of *Paleodictyon* density and distribution at the studied stations.

Station	Lat. (Start/End)	Long. (Start/End)	Depth (m)	Transect distance (m)	Still images (N)	In-focus area (m^2^)	*Paleodictyon* (N)	Density (indiv./m^2^)		
								Combined	PM1	PM2
4	51° 37,726′ N/51° 37,728′ N	170° 28,978′ W/170° 30,082′ W	4607	1270	684	1152	86	0.074	0.020	0.053
6	50° 38,573′ N/50° 40,356′ N	169° 49,165′ W/169° 48,084′ W	5327–5255	3530	1354	3203	0	–	–	–
8	52° 21,955′ N/52° 21,846′ N	167° 05,050′ W/167° 06,373′ W	4609	1510	899	1370	22	0.016	0.006	0.009
10	51° 40,993′ N/51° 40,332′ N	166° 32,591′ W/166° 33,266′ W	5090	1450	863	1315	0	–	–	–
12	53° 35,650′ N/53° 36,293′ N	162° 10,217′ W/162° 10,998′ W	4299	1470	711	1334	214	0.160	0.087	0.072
14	52° 42,799′ N/52° 42,803′ N	161° 49,179′ W/161° 50,496′ W	4872	1470	791	1334	115	0.086	0.073	0.012

**Figure 2:**
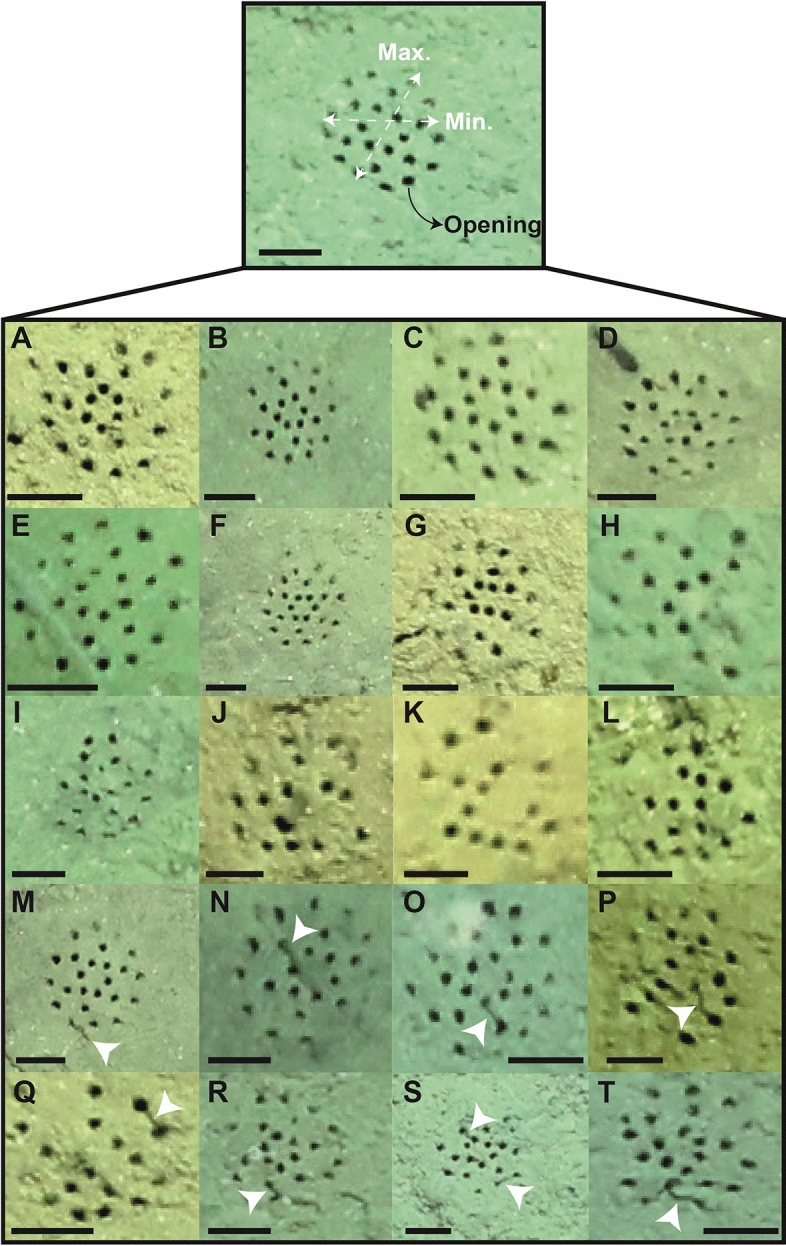
*Paleodictyon* example showing the measured morphological features. Below, representative *Paleodictyon* specimens sighted in the Aleutian Trench stations. (**A-F**) PM1 specimens. (**G**) PM1 with an incomplete inner hexagonal pattern. (**H–L**) PM2 specimens. (**M-T**) *Paleodictyon* specimens with nearby unidentified fauna (white triangles). All scale bars = 1 cm.

Morphological analysis of *Paleodictyon* was based first on visual observation in order to differentiate morphotypes with an inner hexagonal pattern (*Paleodictyon* Morphotype 1; PM1) from those with non-hexagonal patterns making up irregular arrays of openings (*Paleodictyon* Morphotype 2; PM2). The number of openings per specimen and mesh size (the average value between maximum and minimum length of the mesh) were then measured (see Fig. 2). Finally, the ratio between the number of openings and the mesh size (O/S) was calculated to test *Paleodictyon* mesh density. Image measurements were completed in the Open Source software Fiji^18^.

The statistical analysis of *Paleodictyon* morphology consisted of two parts with different objectives. 1) A morphological comparison between the morphotypes based on the above-mentioned parameters. Since the morphological data for PM1 shows a non-normal distribution, a Wilcoxon–Mann–Whitney (WMW) test was used to compare the two morphotypes. 2) An analysis of the variability of the O/S for PM1 and PM2 at different stations using a pairwise Mann–Whitney test. Environmental data layers (e.g., bottom current velocity, temperature, salinity, phosphate, nitrate, silicate, iron, dissolved oxygen and chlorophyll) were used for inter-station correlation and comparison with *Paleodictyon* variability. Environmental data layers were downloaded at a 5 arcmin (c. 9.2 km at the equator) spatial resolution from Bio‐ORACLE v2.0^19^ using the R-packages ‘sdmpredictors’^20^ and ‘raster’^22^ in R Studio^23^. Spearman's rank correlation coefficient was used to test correlation between *Paleodictyon* morphotypes and environmental parameters. All statistical tests were conducted using PAST v. 4.12^24^.

## Results

A total of 437 *Paleodictyon* specimens was observed at stations 4, 8, 12, and 14 (PM1 = 248; PM2 = 189) within a depth range of 4299 to 4872 m (Fig. 1; Supplementary Information). *Paleodictyon* was not observed at stations 6 (5255–5327 m) and 10 (5090 m) (Fig. 1). The density of *Paleodictyon* varied according to the station, reaching a maximum of 0.16 indiv./m^2^ at the shallowest station (station 12) and a minimum of 0.016 indiv./m^2^ at station 8 (Table 1). The density of PM1 becomes higher towards the east while PM2 becomes dominant towards western stations (Fig. 1).

PM1 comprises six central openings arranged in a neat hexagonal circuit with an outer circular to oval array of opening with additional openings distributed between these inner and outer circuits (Fig. 2A–F). Occasional specimens are missing one opening from the hexagonal center, but these are rare, and since they have a similar overall configuration, they have been included within the PM1 group (Fig. 2G)*.* The typical PM1 pattern comprises two concentric hexagonal patterns in the center and a surrounding circle of openings to create a trace with an overall circular to oval shape (Fig. 2D–F). The PM2 traces are characterized by openings that seem to have non-hexagonal distribution (Fig. 2H–L), although some may be aligned in rows (Fig. 2H and K). The average mesh size of the two morphotypes combined is 1.81 cm ± 0.23 cm (max. value 2.51 cm) and the openings diameters are commonly smaller than 2 mm. There is no evidence that either morphotype has a shield-like central mound, although there may be some minor topographic irregularity across the mesh area. There were no obvious animals associated with *Paleodictyon* in the analysed still images. However, in a few cases, dark, elongate structures with a worm-like shape emerge from the openings (Fig. 2M and N). Some are looped into a shape somewhat resembling a caterpillar, while others link two nearby openings (Fig. 2O–T).

The comparison between PM1 and PM2 reveals significant differences in all morphological features (number of openings, mesh size, and O/S) (Table 2). PM1 traces have larger values for O/S and size as well as a considerably higher number of openings (Table 2). The two morphotypes are therefore clearly different rather than being variants of one form. Inter-station analysis shows that PM1 specimens from western stations (stations 4 and 8) have similar O/S values (p = 0.08) while those from eastern stations (stations 12 and 14) show significant variability (all MWM tests have p < 0.01). Among PM2 traces, those from stations 14 and 8 have similar O/S values (p = 0.73), while those from stations 12 and 4 display significant variability (p < 0.01). However, Spearman's rank correlation coefficient does not reveal any environmental parameters (Bio‐ORACLE data layers) that are correlated with the distribution and density of PM1 and PM2 (Table 3). Thus, the driver of this variability remains unclear. Moreover, the correlation of general *Paleodictyon* distribution with Bio‐ORACLE data layers shows that the absence of *Paleodictyon* at stations 6 and 10 is related only to water depth (Table 3).

**Table 2:** Morphological data of *Paleodictyon* species PM1 and PM2 identified from the abyssal area of the Aleutian trench data. Note that all parameters are significantly different. Wilcoxon–Mann–Whitney-test (WMW).

Metric	Morphotype	n	Mean	95% CI	WMW test
Number of openings per *Paleodictyon* morphotypes	PM1	248	20.09	19.66–20.52	** < 0.01**
	PM2	189	16.67	16.14–17.21	
Mesh size *Paleodictyon* (cm)	PM1	248	1.84	1.82–1.87	** < 0.01**
	PM2	189	1.75	1.71–1.78	
Number of openings/mesh size per *Paleodictyon* (n/cm)	PM1	248	10.92	10.69–11.15	** < 0.01**
	PM2	189	9.60	9.27–9.93	

Significant values are in bold.

**Table 3:** Environmental data layers (downloaded from Bio‐ORACLE v2.0^19^) and *Paleodictyon* density (indiv./m^2^) for each species (PM1 and PM2). Note that the only environmental variable that is related with *Paleodictyon* absence in stations 6 and 10 is water depth.

Station	PM1 (indiv./m^2^)	PM2 (indiv./m^2^)	Water depth (m)	Current velocity (m/s)	Temperature (°C)	Salinity (PSS)	Nitrate (µmol/m^3^)	Chlorophyll (mg/m^3^)	Dissolved oxygen (µmol/m^3^)	Silicate (µmol/m^3^)	Iron (µmol/m^3^)	Phosphate (µmol/m^3^)
4	0.02	0.053	−4607	0.0219	1.1088	34.6860	37.4796	0.0044	342.1327	171.2060	0.0006	260.4402
6	0	0	−5327/−5255	0.0352	1.1058	34.6862	37.4947	0.0044	339.6151	172.6347	0.0006	261.1686
8	0.006	0.009	−4609	0.0309	1.1188	34.6860	37.5595	0.0044	340.7840	173.3999	0.0006	260.7863
10	0	0	−5090	0.0364	1.1084	34.6892	37.5034	0.0044	341.4534	173.9635	0.0006	260.9223
12	0.087	0.072	−4299	0.0356	1.1347	34.6868	37.6973	0.0044	337.3358	175.7822	0.0006	261.7667
14	0.073	0.012	−4872	0.0363	1.1167	34.6863	37.5877	0.0044	343.7074	176.5287	0.0006	261.1892

## Discussion

### Paleodictyon diversity and density

Our results demonstrate the existence of two clearly differentiated *Paleodictyon* morphotypes (all WMW tests have a p < 0.01). PM1 has a central hexagonal pattern and slightly larger morphological features (mesh size, number of openings, and O/S ratios) than PM2*,* which is characterized by non-hexagonal distributed openings. Since 32 fossil ichnospecies of *Paleodictyon* have been distinguished based on different sizes and morphologies^3^, modern analogues might be expected to display high diversity as well. In fact, the majority of described specimens have been assigned to the relatively large ichnospecies *Paleodictyon nodosum*, which reaches a size of up to 7.5 cm^5,7,9^. The recent study of Boehringer et al.^8^ is the only one to distinguish two types of modern *Paleodictyon* and analyze them separately. The absence of a greater diversity of modern *Paleodictyon* morphotypes may be related to the morphometrics used. In the present study, O/S was used to check the density of the mesh. In other words, to test if *Paleodictyon* specimens represent a continuum with various degrees of mesh density or whether they are truly distinct morphotypes. This parameter could be a useful tool to differentiate ichnospecies of *Paleodictyon* and reveal overlooked diversity.

Both of our morphotypes displayed significant differences in their morphological characteristics and are much smaller than any previously reported specimens worldwide and also have smaller size variability. Thus, both Aleutian morphotypes are best considered as different ichnospecies of *Paleodictyon* and certainly distinct from *P*. *nodosum*. However, although PM1 and PM2 are different ichnospecies, it is not possible to establish whether they are produced by the same organism since the identity of the tracemaker is unknown. Also, no environmental factor has been identified to explain their density variation among stations (Table 3). We hope that improvements in seafloor observation technology will lead to the recognition of a greater diversity among *Paleodictyon* as well as shedding some light on the environmental parameters controlling *Paleodictyon* diversity. However, for the present, the question of what factors influence the diversity of *Paleodictyon* remains.

The density of *Paleodictyon* in the study area was considerably lower than the maximum densities (45 indiv./m^2^)found at the MAR^5^ or along the Indian Ridge (9.7 indiv./m^2^), where patchy distributions were observed^9^. Also, the densities in the Aleutian area were three orders of magnitude lower than the average values reported for the CCZ (0.3 indiv./m^2^ in Durden et al.^7^; and 0.2 indiv./m^2^ in Boehringer et al.^8^) but higher than those reported in the DISCOL Experimental Area (DEA) in the Southeastern Pacific (0.0033 indiv./m^2^ in Boehringer et al.^8^). Mesh sizes (usually referred as diameter) reported in the literature span from 1.8 to 7.5 cm, with the average size always being more than 2.5 cm^5–9^. The Aleutian examples are considerably smaller than the previously published examples, except for some specimens from the DEA (Fig. 2e in Boehringer et al.^8^).

### Controls on Paleodictyon morphology and distribution

Various environmental factors have been suggested to explain *Paleodictyon* distribution, density and size. On the Mid-Atlantic Ridge, where modern examples were first discovered, the traces are confined to sites with low sedimentation rates, but as Durden et al.^7^ indicated, they have occurred in areas of higher sedimentation, while absent in areas with lower sedimentation rates. It has also been suggested that trends in *Paleodictyon* densities, as well as the size of individual patterns, may be related to their distance from hydrothermal areas, with lower densities on non-vent plains and *vice versa*^5,9^. These relationships could be linked to higher food availability around vents^9^. However, Atlantic and Pacific abyssal plains, where there is no hydrothermal activity, host *Paleodictyon* of equal size and in similar densities^6,7^. *Paleodictyon* are always associated with bottom water that is well-oxygenated and relatively cold. The Atlantic has somewhat warmer and better oxygenated bottom water than the Pacific^24^, but this does not seem to influence *Paleodictyon* distributions since similar *P. nodosum* morphotypes are found at temperate latitudes in both oceans. All previous modern examples of *Paleodictyon* were restricted to tropical/subtropical latitudes with a maximum water depth of 4189 m in the DEA^5,9^, whereas our Aleutian specimens occurred at Subarctic latitudes (51°–53°N) at a depth of over 4500 m. Thus, our results show that the occurrence of *Paleodictyon* is not limited by latitude, whereas its absence at stations 6 and 10 (the only two deeper than 5000 m) suggests that water depth may be a limiting factor for the tracemaker. Also, a consistent environmental requirement for *Paleodictyon* appears to be the presence of soft, fine-grained sediments^5,9,14^. Traces have been observed even when fine sediments are associated with hard substrates, for example, polymetallic nodule fields in the equatorial Pacific where nodules have been observed to interrupt *Paleodictyon* patterns^7,8^, or patchy sediment overlying a basalt substrate in the central Indian Ocean^9^. The sediments are usually calcareous *Globigerina* ooze, but the Aleutian traces occur on siliceous oozes with radiolarians and diatoms (A.J. Gooday per. obs.). In short, based on the currently known worldwide *Paleodictyon* distribution, apart from water depth, controls on the density and distribution of traces remain unclear.

**Figure 3:**
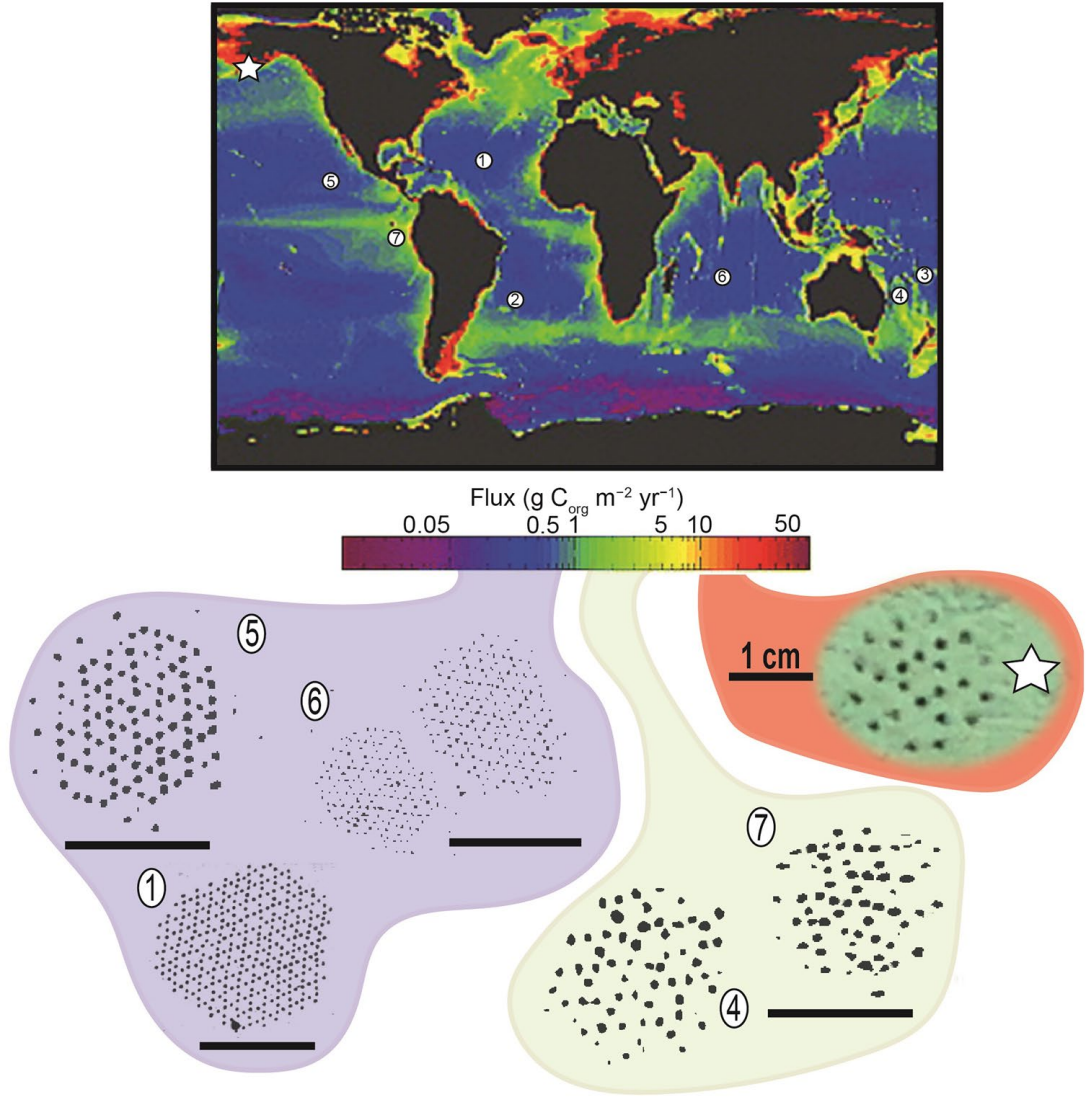
Map of the global ocean forecasts of annual average particulate organic carbon flux to the seafloor (g C_org_ m^−2^ yr^−1^; modified from Fig. 14d in Lutz et al.^31^) and the worldwide distribution of *Paleodictyon* discoveries. Below, *Paleodictyon* sketches from each locality (sketches based on illustrated examples in the literature^5–9^) and inferred particulate organic carbon flux to the seafloor areas. Purple examples are assigned to *P*. *nodosum*. Scale bars = 5 cm (except for the studied specimen). Note the considerable smaller size of the Aleutian specimen.

Food limitation characterizes much of the deep sea, particularly at abyssal depths^25^. As a result, particulate organic matter (POC) fluxes to the ocean floor are thought to be the main drivers of many ecological processes and benthic community attributes such as respiration, bioturbation, and the abundance and biomass of different faunal compartments^25–27^. On a global scale, POC flux was one of the main factors used by Watling et al.^24^ to define faunal provinces at lower bathyal and abyssal depths. At local and regional scales, a clear relationship may exist between the abundance of particular species and POC fluxes, for example, among the Foraminifera, a group for which a considerable body of species-level data exists^28–30^. Given these considerations, it is perhaps not surprising that there also appears to be a relationship between POC fluxes^31^ and the distribution of *Paleodictyon* morphotypes. As shown in Fig. 3, larger, well-organised patterns resembling *P. nodosum* are associated with lower fluxes (Mid-Atlantic Ridge, CCZ and Indian Ocean; 0.5–1 g C_org_ m^−2^ yr^−1^), more disorganized patterns associated with moderate fluxes (DEA, Eastern Australian margin; −5 g C_org_ m^−2^ yr^−1^), while the relatively small Aleutian traces are associated with higher fluxes (> 10 g C_org_ m^−2^ yr^−1^; Fig. 14d in Lutz et al.^31^). This suggests that the Aleutian examples are produced by a distinct tracemaker that is adapted to more eutrophic conditions than previously described forms.

The fact that the Aleutian *Paleodictyon* are smaller and have fewer openings that those from lower latitudes may have autecological as well as taxonomic significance. Since these traces have no shield-mound to induce effective ventilation through the openings^5,16^, the tracemakers presumably rely on organic matter that reaches the seafloor and falls inside the openings, or that they actively collect. This might only be a viable strategy in areas where more food is available. In more oligotrophic regions, the tracemaker may require a wider mesh in order to maximise organic matter capture. Moreover, these larger traces are usually associated with a shield mound that can enhance burrow ventilation and food acquisition (see Figs. 11 and 12 in Rona et al.^5^). This suggests that *Paleodictyon* morphology reflects the behavioural response of the tracemaker to the environmental conditions. Thus, *Paleodictyon* size may be a good indicator of food availability in the fossil record. It has also been suggested that *Paleodictyon* tracemakers, as well as those of other complex graphoglyptid traces, have an agrichnial gardening strategy that involves the culturing of microorganisms within the burrow system as a food source^2,32–34^. This interpretation has been controversial (see discussion by Hsieh et al.^35^), but if correct, then a larger burrow wall area would provide more space in which to culture bacteria, an advantage in more oligotrophic settings but less advantageous where more food is available and a smaller mesh will suffice.

## Conclusions

We describe two *Paleodictyon* morphotypes from abyssal depths near the Aleutian Trench. Both are smaller and morphologically different from previously reported specimens. This is the first record of *Paleodictyon* at Subarctic latitudes (51°–53°N) and below 4500 m depth. Our results lead to the following conclusions:

There is no obvious correlation between environmental factors and the distribution of the morphotypes, both of which occur together at all sites. However, their absence at the deepest stations (> 5000 m) may indicate that some water depth-related factor is limiting their occurrence.

The ratio between the number of openings and the mesh size seems to be a good parameter for differentiating *Paleodictyon* morphotypes and exploring their diversity in modern oceans. The Aleutian *Paleodictyon* are clearly different from previously analyzed specimens and seem to represent different ichnospecies, whose tracemakers are adapted to the more eutrophic conditions prevailing at these northerly latitudes.

At a global scale, *Paleodictyon* size (i.e., mesh size and number of openings) seems to be correlated with the POC flux to the seafloor. The fact that smaller *Paleodictyon* morphotypes are associated with more eutrophic environments, and vice versa, suggests that the size of the traces may be used as an indicator of nutrient fluxes in the fossil record.

Finally, the mystery surrounding the *Paleodictyon* tracemakers remains a challenge for the future. A few of the more than 400 specimens analyzed during the present study show some sort of “tentacle-like” structures associated with the openings. Unfortunately, the resolution of the images is not sufficient to determine the nature of these intriguing features, or whether they have any bearing on the identity of the organism responsible for the traces.

## Supplementary Information

Supplementary Information.

## Data Availability

All data generated or analysed during this study are included in this published article. The raw data used for this study is in the Supplementary Information file.
